# Identification and Functional Characterization of the Axolotl (*Ambystoma mexicanum*) Tau Protein

**DOI:** 10.1002/alz70855_103601

**Published:** 2025-12-26

**Authors:** Zachary Strickland, Benoit Giasson, Jada Lewis

**Affiliations:** ^1^ University of Florida, Gainesville, FL, USA

## Abstract

**Background:**

Tauopathies are characterized by abnormal aggregation of tau that results in neuronal dysfunction and degeneration. Developing strategies to preserve existing neuronal function, prevent neurodegeneration, or restore lost neurons may have the most potential to combat human tauopathy; however, the adult human brain possesses limited capacity to replace lost neurons. Interestingly, some vertebrate species such as axolotls (*Ambystoma mexicanum*) possess the capacity to regenerate large portions of their anatomy, including the brain and spinal cord. We previously reported that axolotls express a tau protein with high similarity to human tau in the putative microtubule binding domain and C‐terminus. In humans, missense mutations of the *MAPT* gene at the Proline‐301 residue within the microtubule binding domain to a lysine (P301L) or serine (P301S) increase the self‐aggregation ability of tau and are causal for tauopathy.

**Methods:**

We previously compared the degree of homology between the human, axolotl, and mouse tau gene and protein. Using confocal immunofluorescent microscopy, we studied co‐localization between axolotl tau and the microtubule subunit protein β‐tubulin III. We will perform proximity ligation assays in fixed axolotl brain tissues to further validate proximal interactions between axolotl tau and β‐tubulin III. Utilizing *in vitro* tau self‐aggregation assays and tau‐microtubule binding assays, we will determine axolotl tau's ability to aggregate and bind microtubules. As the axolotl tau microtubule binding domain possesses a Proline‐301 equivalent site (Proline‐387), we will also explore how P387S and P387L mutations change the aggregation potential and microtubule binding ability of axolotl tau.

**Result:**

We will report the first ever characterization of axolotl tau's ability to bind to microtubules and potential for self‐aggregation and how mutations equivalent to the human P301L and P301S tau mutants impact these abilities.

**Conclusion:**

Our microscopy data suggests that tau and β‐tubulin III co‐localize in axolotl brain tissue. These results will further elucidate our understanding of tau‐microtubule interactions in the axolotl, unveil axolotl tau's capacity for aggregation, and position the axolotl as a novel model system in which to investigate how a regenerative organism might respond to an attempt to induce tauopathy.

